# PCB-95 Promotes Dendritic Growth via Ryanodine Receptor–Dependent Mechanisms

**DOI:** 10.1289/ehp.1104832

**Published:** 2012-04-25

**Authors:** Gary A. Wayman, Dongren Yang, Diptiman D. Bose, Adam Lesiak, Veronica Ledoux, Donald Bruun, Isaac N. Pessah, Pamela J. Lein

**Affiliations:** 1 Program in Neuroscience, Department of Veterinary and Comparative Anatomy, Pharmacology and Physiology, Washington State University, Pullman, Washington, USA; 2 Department of Molecular Biosciences, University of California, Davis, Davis, California, USA; 3 Center for Research on Occupational and Environmental Toxicology, Oregon Health & Science University, Portland, Oregon, USA

**Keywords:** dendrites, developmental neurotoxicity, hippocampal neurons, neuronal connectivity, non-dioxin-like PCBs, ryanodine receptor

## Abstract

Background: Aroclor 1254 (A1254) interferes with normal dendritic growth and plasticity in the developing rodent brain, but the mechanism(s) mediating this effect have yet to be established. Non-dioxin-like (NDL) polychlorinated biphenyls (PCBs) enhance the activity of ryanodine receptor (RyR) calcium ion (Ca^2+^) channels, which play a central role in regulating the spatiotemporal dynamics of intracellular Ca^2+^ signaling. Ca^2+^ signaling is a predominant factor in shaping dendritic arbors, but whether PCB potentiation of RyR activity influences dendritic growth is not known.

Objective: We determined whether RyR activity is required for PCB effects on dendritic growth.

Methods and Results: Golgi analysis of hippocampi from weanling rats confirmed that developmental exposure via the maternal diet to NDL PCB-95 (2,2´,3,5´6-pentachlorobiphenyl), a potent RyR potentiator, phenocopies the dendrite-promoting effects of A1254. Dendritic growth in dissociated cultures of primary hippocampal neurons and in hippocampal slice cultures is similarly enhanced by PCB-95 but not by PCB-66 (2,3,4´,4-tetrachlorobiphenyl), a congener with negligible effects on RyR activity. The dendrite-promoting effects of PCB-95 are evident at concentrations as low as 2 pM and are inhibited by either pharmacologic blockade or siRNA knockdown of RyRs.

Conclusions: Our findings demonstrate that environmentally relevant levels of NDL PCBs modulate neuronal connectivity via RyR-dependent effects on dendritic arborization. In addition, these findings identify RyR channel dysregulation as a novel mechanism contributing to dysmorphic dendritogenesis associated with heritable and environmentally triggered neurodevelopmental disorders.

Dendritic architecture is a critical determinant of neuronal connectivity (Libersat and Duch 2004; Scott and Luo 2001). Abnormalities in dendritic shape are the most consistent pathologic correlate of behavioral deficits in heritable and environmentally triggered neurodevelopmental disorders (Bourgeron 2009; Fukuda et al. 2005; Garey 2010; Penzes et al. 2011; Svitkina et al. 2010). Polychlorinated biphenyls (PCBs) are known human developmental neurotoxicants (Carpenter 2006; Korrick and Sagiv 2008; Schantz et al. 2003), and we previously demonstrated that developmental exposure to the PCB mixture Aroclor 1254 (A1254) interferes with normal patterns of dendritic growth and plasticity in weanling rats (Lein et al. 2007; Yang et al. 2009) coincident with deficits in spatial learning and memory (Yang et al. 2009).

The dynamic structural remodeling of dendrites that occurs during development (Chen and Nedivi 2010; Cline 2001) is driven in large part by calcium ion (Ca^2+^)-dependent signaling pathways triggered by NMDA (*N*-methyl-d-aspartate) receptor activation and extrinsic cues such as neurotrophins (Lohmann and Wong 2005; Wayman et al. 2008). Non-dioxin-like (NDL) PCBs increase intracellular Ca^2+^ in neurons via several mechanisms, the most sensitive of which is potentiation of ryanodine receptor (RyR) activity (Pessah et al. 2010). RyRs are ion channels in the endoplasmic reticulum (ER) that regulate Ca^2+^ release from the ER and modulate the gating response and signal gain of plasma membrane ion channels, including the NMDA receptor (Pessah et al. 2010). RyR activity determines the amplitude and spatiotemporal patterns of intracellular Ca^2+^ fluxes (Berridge 2006). NDL PCBs interact with RyRs to dramatically alter their sensitivity to physiological modulators (Wong et al. 1997).

A1254 consists primarily of NDL PCBs (Kostyniak et al. 2005) with varying RyR-sensitizing potencies (Pessah et al. 2006), suggesting that effects of developmental A1254 exposure on dendritic arborization are mediated by RyR-dependent mechanisms. In support of this hypothesis, we demonstrated that A1254 interference with dendritic growth and plasticity in cerebellar Purkinje cells correlated with altered RyR expression and activity in the cerebellum (Yang et al. 2009) and that nanomolar concentrations of environmentally relevant PCB-95 (2,2´,3,5´,6-pentachlorobiphenyl), a congener that potently activates RyRs (Pessah et al. 2006), enhanced dendritic growth in primary cultures of neocortical neurons (Yang et al. 2009). Here, we provide evidence of a causal relationship between PCB-95–induced dendritic growth and RyR activation in hippocampal neurons.

## Methods

A complete listing of reagents is provided in Supplemental Material, p. 3 (http://dx.doi.org/10.1289/ehp.1104832). Animals were treated humanely and with regard for alleviation of suffering according to protocols approved by the Institutional Animal Care and Use Committees of the Johns Hopkins University; Oregon Health & Science University; University of California, Davis; and Washington State University, Pullman.

In vivo *PCB exposures.* Adult Long Evans rats were purchased from Charles River Laboratories (Hollister, CA), and husbandry practices were as previously described (Yang et al. 2009). Dams were dosed with A1254 (1 or 6 mg/kg/day), PCB-95 (0.1, 1, or 6 mg/kg/day), or vehicle (peanut oil) as previously described (Yang et al. 2009) beginning 14 days before breeding and continuing until postnatal day (PD) 21. Dams delivered 10–15 pups (*n* = 11 dams per treatment group). By PD2, litters were culled to 10 pups. Pups were weaned on PD21 and euthanized at PD31 (A1254 studies) or PD38 (PCB-95 studies). Exposure to either A1254 or PCB-95 in the maternal diet throughout gestation and lactation did not affect the body weight of pregnant and lactating dams, litter size, sex ratios, or growth rates of the pups.

*Cell culture.* Hippocampal neurons (10^5^ cells/cm^2^) were dissociated from PD1 Sprague-Dawley rats (Charles River Laboratories) and cultured in Neurobasal-A (Invitrogen, Carlsbad, CA) supplemented with B27 (Invitrogen) as described previously (Wayman et al. 2006). At 5–6 days *in vitro* (DIV), cultures were transfected with plasmid-encoding microtubule-associated-protein-2B (MAP2B) fused to enhanced green fluorescent protein (EGFP), which selectively labels the somatodendritic domain (Wayman et al. 2006), using Lipofectamine-2000 (Invitrogen) according to the manufacturer’s protocol. At 7 DIV, cultures were treated for 48 hr with vehicle (DMSO; 1:1,000 dilution), PCB-95 (2 fM–2 μM), or PCB-66 (2,3,4´,4-tetrachlorobiphenyl; 200 nM) diluted from 1,000× stocks. Hippocampal slices from PD5 Sprague-Dawley rats were cultured for 3 days as described (Lein et al. 2011). To visualize dendritic arbors, slice cultures were biolistically transfected with plasmid-encoding tomato fluorescent protein (TFP), which fills the entire cell, using a Helios Gene Gun (BioRad, Hercules, CA), according to the manufacturer’s protocol. DNA amounts, transfection reagent amounts, and transfection duration were optimized to minimize toxicity and maximize transfection efficiency. Following transfection, slices were allowed to recover for 24 hr before exposure to PCB-95 for 48 hr.

*Dendritic analyses.* Dendritic arbors of pyramidal neurons in the CA1 hippocampus of weanling rats were Golgi stained and quantified by Sholl analysis (Lein et al. 2007). Sholl data were evaluated using the Wilcoxon rank-sign test applying a conservative alpha level based on the number of measurements (Dawson and Trapp 2004). Soma size was analyzed using Image J version 1.44p with the Neuron J plug-in version 1.42 to trace neurons (Meijering et al. 2004), and significant differences were determined using Student *t*-test with significance set at *p* < 0.05. Dendritic morphology in dissociated hippocampal cultures or hippocampal slice cultures was quantified from digital images of green fluorescent protein–positive (GFP^+^) or TFP^+^ neurons, respectively, using NeuronJ (Meijering et al. 2004). Dendritic length and number of dendritic termini per neuron were analyzed by one-way analysis of variation with significance set at *p* < 0.05. Differences between treatment groups were identified by *post hoc* Tukey’s test. All morphometric experiments were replicated in cultures derived from at least three independent dissections.

*RyR expression.* RyR expression in lysates (25 µL/well) of 2-, 7-, and 12-DIV dissociated cultures or whole membrane (100,000 × *g*) fractions prepared from adult (2–4 month old) rat hippocampi were size-separated on SDS-PAGE (4–20%) and Western blotting was performed using anti-RyR1 antibody 34C (1:500; University of Iowa Hybridoma Bank, Iowa City, IA) or anti-RyR2 antibody C3-33 (1:500; generous gift of G. Meissner, University of North Carolina–Chapel Hill) as previously described (Yang et al. 2009). Blots were re-probed with a monoclonal antibody (Sigma-Aldrich Corp., St. Louis, MO) that recognizes α-tubulin to which RyR bands were normalized. To localize RyR in hippocampal neurons, dissociated cultures were fixed in 4% paraformaldehyde at 21 DIV, permeabilized with 0.05% Triton X-100, blocked in PBS (phosphate buffered solution) containing 2% glycerol, 0.05 M NH_4_Cl (ammonium chloride), 5% FBS (fetal bovine serum), 2% goat serum and then incubated with fluorochrome-tagged phalloidin (Molecular Probes, Invitrogen; 1:200) and antibody C3-33 (1:100), which recognizes all three RyR isoforms at that dilution (Meissner 2002). Antibody–antigen complexes were visualized using fluorochrome-conjugated secondary antibodies (Molecular Probes, Invitrogen). Z-stack images were obtained using a Delta Vision Core Imaging System (Applied Precision, LLC, Seattle, WA) and deconvoluted using Imaris software, version 6.2 (Bitplane, South Windsor, CT), as previously described (Chen et al. 2010).

## Results

**Figure 1:**
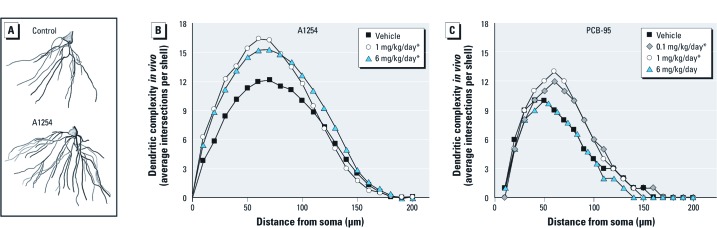
PCBs enhance dendritic growth *in vivo*. (*A*) Representative camera lucida images and (*B*) Sholl analyses of the basilar dendritic arbor of Golgi-stained CA1 pyramidal neurons from PD31 weanling rats exposed throughout gestation and lactation to vehicle or A1254 in the maternal diet (*n* = 45 neurons per group). (*C*) Sholl analyses of the basilar dendritic arbor of Golgi-stained pyramidal neurons from PD38 weanling rats exposed throughout gestation and lactation to vehicle or PCB-95 in the maternal diet (*n* = 60 neurons per group). *p < 0.05 relative to vehicle control.

*PCBs enhance dendritic growth in the developing hippocampus.* A1254 is a commercial PCB mixture that includes many of the congeners associated with human exposures (Kodavanti et al. 2001; Kostyniak et al. 2005). We have previously showed that exposure to A1254 at 1 and 6 mg/kg/day in the maternal diet throughout gestation and lactation resulted in PCB levels in the brains of weanling rats comparable to those reported in human brain tissue and also promoted dendritic growth in cerebellar Purkinje cells and neocortical pyramidal neurons (Yang et al. 2009). Using this dosing regimen, we examined the effects of developmental A1254 exposure on hippocampal pyramidal neurons at PD31. Representative camera lucida drawings of the basilar dendritic arbor of Golgi-impregnated CA1 pyramidal neurons demonstrate that A1254 significantly increases dendritic complexity (Figure 1A). Sholl analysis indicates that dendritic arborization was significantly increased relative to vehicle controls by 25% and 29% in the 1 and 6 mg/kg/day A1254 treatment groups, respectively (Figure 1B).

To determine the potential contribution of RyR-active NDL PCBs to the dendrite-promoting activity of A1254, we examined dendritic arborization in pyramidal neurons of weanling rats exposed throughout gestation and lactation to PCB-95 in the maternal diet at 0.1, 1.0, or 6.0 mg/kg/day. PCB-95 is the most potent RyR activator yet identified among the NDL congeners (Pessah et al. 2006). Sholl analyses of Golgi-stained pyramidal neurons in PD38 weanlings indicated that developmental PCB-95 exposure increased dendritic growth by 20% and 21% in the 0.1 and 1.0 mg/kg/day PCB-95 treatment groups, respectively, but that dendritic growth in the 6 mg/kg/day PCB-95 treatment group did not differ significantly from vehicle controls (Figure 1C). Consistent with previous studies (Lein et al. 2007), soma size of pyramidal neurons was not affected by developmental exposure to either A1254 or PCB-95 (data not shown).

**Figure 2:**
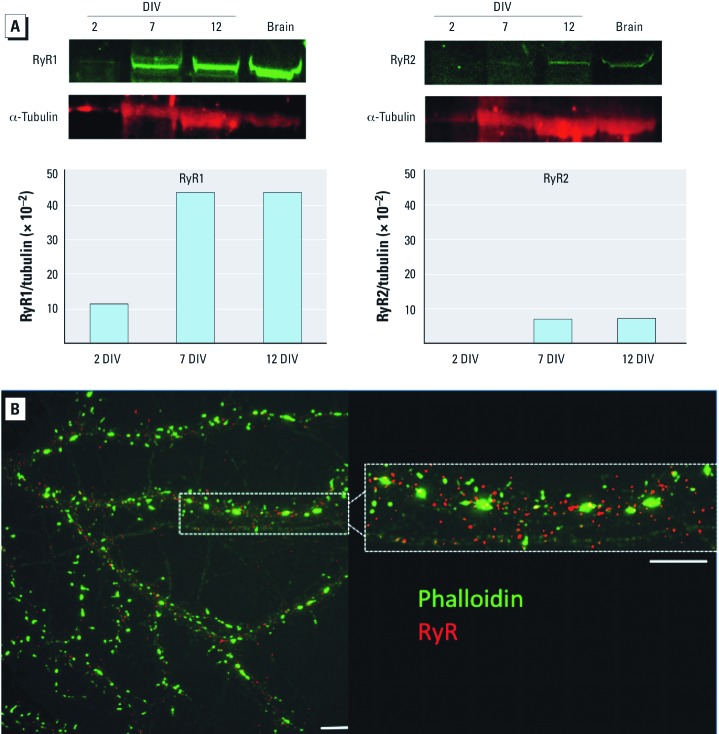
RyR expression in cultured hippocampal neurons. (*A*) Representative blots and densitometric analyses of membrane fractions from rat hippocampal cultures harvested at 2, 7, and 12 DIV, and probed with RyR1- or RyR2-selective monoclonal antibodies and a monoclonal antibody for α-tubulin. Densitometric data are presented as the pixel intensity of the RyR-immunoreactive band normalized to the pixel intensity of the α-tubulin immunoreactive band in the same sample. Expression of RyR1 and RyR2 at 7 and 12 DIV was verified in cultures derived from two separate dissections. “Brain” indicates membranes prepared from adult (2- to 4-month-old) mouse hippocampi. (*B*) Fluorescent photomicrograph illustrating subcellular localization of RyR immunoreactivity (red) and phalloidin reactivity (green) in a 21-DIV mouse hippocampal neuron. Bar = 5 μM.

*RyR expression in cultured hippocampal neurons.* Mechanistic studies of RyR involvement in the dendrite-promoting activity of PCB-95 were studied with primary cultured hippocampal neurons. All three RyR isoforms are expressed in the brain although their relative expression varies as a function of developmental age and brain region (Berridge 2006); therefore, we first determined patterns of RyR expression in cultured hippocampal neurons by Western blotting. Monoclonal antibody (mAb) 34C or C3-33 at concentrations that selectively bind to RyR1 and RyR3 (Airey et al. 1990) or to RyR2 (Lai et al. 1992), respectively labeled 565 and 557 kDa bands, in 7- and 12-DIV lysates, whereas only RyR1 was detectable at 2 DIV (Figure 2A). A band with a lower molecular mass corresponding to RyR3 (545 kDa) was not detected by mAb 34C from cultures at any time point examined. This is consistent with reports that RyR3 accounts for about 2% of the total RyR protein in the brain (Kim et al. 2007; Murayama and Ogawa 1996) and in hippocampal cultures not stimulated with BDNF (brain-derived neurotrophic factor) (Adasme et al. 2011). These data verify that both RyR1 and RyR2 were expressed in hippocampal neurons during the period of most robust dendritic growth in these cultures (Wayman et al. 2006).

Immunocytochemical localization of RyRs in dissociated hippocampal neuronal cell cultures co-labeled with the F-actin label phalloidin to identify actin-rich dendritic spines confirmed RyR immunoreactive puncta throughout the dendritic shafts of cultured hippocampal neurons (Figure 2B).

**Figure 3:**
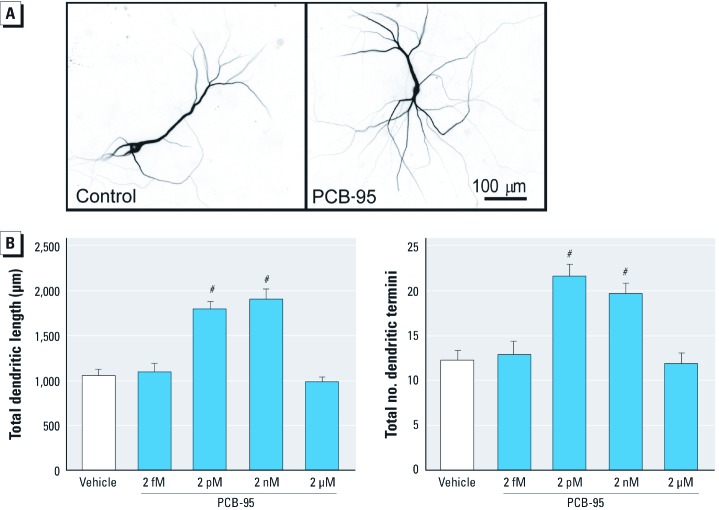
PCB-95 enhances dendritic growth in cultured hippocampal neurons. (*A*) Representative photomicrographs of 9-DIV hippocampal neurons expressing MAP2B-EGFP after 48-hr exposure to vehicle (control) or PCB-95 (2 nM). (*B*) Concentration-dependent effect of PCB-95 on dendritic length and branching (determined as the number of dendritic termini per neuron) in GFP^+^ neurons. Data presented as mean ± SE (*n* = 30 neurons from three to four independent cultures per group). ^#^*p* < 0.001 relative to vehicle control.

*PCB-induced dendritic growth is mediated by RyR-dependent mechanisms.* To further test the validity of our *in vitro* model system, we determined whether the dendrite-promoting activity of PCB-95 is recapitulated in cultured hippocampal neurons. Dendritic arbors of individual neurons in high-density neuron-glia co-cultures were visualized by expressing a MAP2B-EGFP construct under the control of the *CAG* promoter, which exhibits neuron-specific expression (Wayman et al. 2006). Expression of MAP2B-EGFP is restricted to the somatodendritic compartment in cultured hippocampal neurons and does not alter their intrinsic dendritic growth patterns (Wayman et al. 2006). Exposure to PCB-95 between 7–9 DIV significantly enhanced dendritic growth of hippocampal neurons in these cultures as observed at 9 DIV (Figure 3A). Initial concentration range-finding studies indicated increased dendritic length and branching at concentrations as low as 2 pM; however, these effects were no longer evident at concentrations ≥ 2 μM (Figure 3B). Experiments to refine the upper range of the concentration–effect curve indicates dendritic responses of comparable magnitude at concentrations of 2–200 nM; at concentrations of > 200 nM, PCB-95 no longer elicited increased dendritic growth (data not shown). Subsequent mechanistic studies used the maximally effective concentration of 200 nM PCB-95.

**Figure 4:**
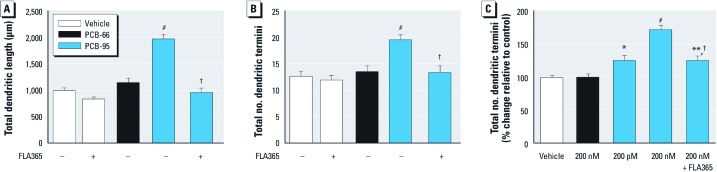
*In vitro* PCB-induced dendritic growth requires RyR activity. PCB-95 (200 nM), but not PCB-66 (200 nM), increased dendritic length (*A*) and branching (*B*) in primary cultures of dissociated hippocampal neurons (9 DIV), and this effect was blocked by the RyR antagonist FLA365 (10 µM) (–, without, +, with FLA365). (*C*) PCB-95, but not PCB-66, significantly enhanced dendritic arborization of TFP^+^ neurons in hippocampal slice cultures, and this effect was blocked by FLA365. Data presented as mean ± SE (*n* = 35–50 neurons from three to four independent cultures per group). *p < 0.05, **p < 0.01, and ^#^*p* < 0.001 relative to vehicle control. ^†^*p* < 0.01 relative to PCB-95 in the absence of FLA365.

If PCBs (200 nM) enhance dendritic growth by potentiating RyR activation, then congeners with differential effects on RyR activation should differentially influence dendritic growth. To test this, we compared dendritic growth in hippocampal cultures exposed to PCB-95 vs. PCB-66, which exhibit potent vs. negligible effects on RyR activity, respectively (Pessah et al. 2006). Exposure to PCB-95 (200 nM) during 7–9 DIV significantly increased total dendritic length and branching as observed at 9 DIV (Figure 4A, B). In contrast, dendritic length and branching in hippocampal neurons exposed to PCB-66 (200 nM) for the same period of time did not differ from that observed in vehicle controls (Figure 4A,B). Moreover, block of RyR channels by FLA365 (4-(2-aminopropyl)-3,5-dichloro-*N*,*N*-dimethylaniline) (Chiesi et al. 1988; Mack et al. 1992) suppressed the dendrite-promoting activity of PCB-95 but had no effect on dendritic length or branching in control cultures not exposed to PCB-95 (Figure 4A,B).

The physiological relevance of these observations was confirmed using hippocampal slice cultures from PD5 rats biolistically labeled with TFP at 3 DIV and then exposed to PCBs from 5–7 DIV. PCB-95, but not PCB-66, significantly increased the number of dendritic termini per neuron in TFP^+^ neurons in a concentration-dependent manner, and this effect was blocked by FLA365 (Figure 4C).

**Figure 5:**
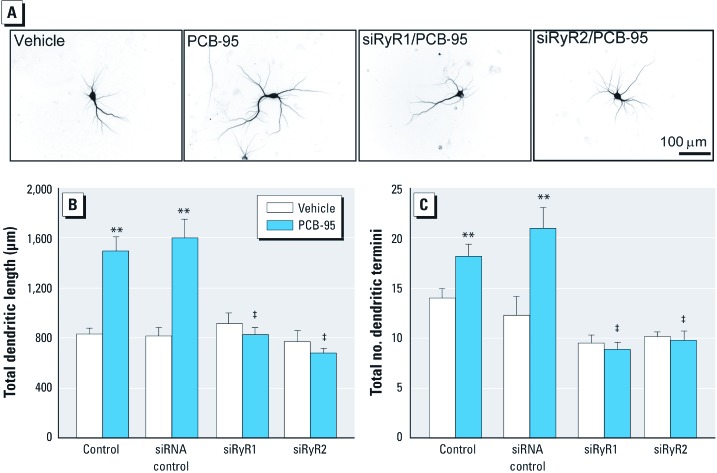
RyR-specific siRNA blocks PCB-induced dendritic growth *in vitro*. (*A*) Representative photomicrographs of 9-DIV hippocampal neurons transfected with MAP2B-EGFP alone or in combination with RyR siRNA before 48-hr treatment with vehicle or PCB-95 (200 nM). Expression of RyR1- or RyR2-specific siRNA, but not scrambled siRNA, blocked PCB-95 effects on dendritic length (*B*) and branching (*C*). Data presented as mean ± SE (*n* = 30 neurons from three independent cultures per group). **p < 0.01 relative to vehicle control. ^‡^*p* < 0.001 relative to untransfected (control) PCB-95–treated hippocampal neurons.

PCB-95 sensitizes both RyR1 and RyR2 (Wong and Pessah 1996). Therefore, to assess the relative contributions of RyR1 and RyR2 in PCB-induced dendritic growth, we expressed siRNA (small interfering RNA) constructs that selectively suppress each RyR isoform [see Supplemental Material, Table S1 (http://dx.doi.org/10.1289/ehp.1104832)]. Experiments in HEK293 cells (see Supplemental Material, p. 4) confirmed that each siRNA blocked expression of its target RyR isoform without altering expression of the other non-target isoform (see Supplemental Material, Figure S1). Expression in hippocampal neurons of siRNA against either RyR1 or RyR2 suppressed PCB-95 stimulation of dendritic length and branching but had no effect on basal dendritic growth (Figure 5). In contrast, expression of control siRNA had no effect on either PCB-95–induced or basal dendritic growth (Figure 5).

## Discussion

We previously reported that developmental A1254 exposure promotes dendritic growth in cerebellar Purkinje cells and cortical pyramidal neurons of weanling rats and that nanomolar concentrations of PCB-95 promote dendritic growth in cultured neocortical neurons (Yang et al. 2009). Here, we extend these observations to show that *a*) developmental exposure to the NDL PCB-95 in the maternal diet phenocopies the effect of developmental A1254 exposure on dendritic arborization in the developing hippocampus, *b*) PCB-95 promotes dendritic growth in cultured hippocampal neurons at picomolar to nanomolar concentrations, and *c*) the dendrite-promoting activity of PCB-95 requires RyR activity.

As a first test of the cause–effect relationship between PCB-enhanced RyR activity and PCB-induced dendritic growth, we compared dendritic growth in cultured hippocampal neurons exposed to PCB-95 vs. PCB-66. PCB-95 is a tri-*ortho* substituted congener that potently sensitizes the RyR channel to physiological and pharmacological activators (Wong and Pessah 1996) and preferentially stabilizes the RyR in its full open conformation (Samso et al. 2009). PCB-66 has a single *ortho*-chlorine substitution, and despite similar physicochemical properties to PCB-95, has negligible influence on RyR activity (Pessah et al. 2006). In cultured hippocampal neurons, PCB-95 promotes dendritic growth, whereas PCB-66 has no effect at the concentrations tested. As a second test, blocking RyR function by either pharmacological antagonism or siRNA knockdown prevented PCB-95–induced dendritic growth. Interestingly, RyR1 siRNA and RyR2 siRNA were equally effective in blocking the dendrite-promoting activity of PCB-95. Studies in HEK cells confirmed that each siRNA was specific for its target mRNA and did not cross-react with non-target RyR mRNA. The native activities of both RyR isoforms are therefore required for the dendrite-promoting activity of PCB-95, and interference with either isoform is sufficient to prevent the influence of RyR-active PCBs, without altering basal dendritic growth or cell viability. The biology underlying the dual requirement for RyR1 and RyR2 in PCB-induced dendritic growth remains to be determined. We see three possible explanations.

One possibility is that each isoform regulates a complementary but distinct profile of downstream effectors necessary for dendritic growth. For example, both transcription- and translation-dependent pathways mediate activity-dependent dendritic growth (Schratt et al. 2004; Tsokas et al. 2005; Vickers et al. 2005; Wayman et al. 2008) and activity-dependent spine formation requires turning on signaling pathways that promote spine formation coincident with turning off signaling pathways that inhibit spine formation (Impey et al. 2010; Saneyoshi et al. 2010).

A second possibility is that activation of spatially segregated RyR1 and RyR2 channels creates Ca^2+^ microdomains within the soma and the dendritic processes and terminals (Berridge 2006) whose coincident activation is necessary for enhancing activity-dependent dendritic growth. As described in the companion paper (Wayman et al. 2012), PCB-95 enhances spontaneous Ca^2+^ oscillations within the soma and distal dendrites of the same neuron, and these effects are blocked by ryanodine. Collectively, these data indicate that NDL PCBs mediate the gain of RyR function that promotes dendritic growth, and they suggest a role for RyR in normal activity-dependent dendritic growth.

The human health relevance of these *in vitro* mechanistic studies is supported by our observations that developmental PCB exposure similarly promotes dendritic growth in the developing brains of weanling rats. Using an exposure paradigm relevant to human PCB exposures in terms of route of exposure, dose level, and dose duration (Yang et al. 2009), we observed that A1254 increases dendritic growth in pyramidal neurons of the CA1 hippocampus. A1254 is composed predominantly of NDL PCB congeners with RyR activity (Kostyniak et al. 2005), consistent with our *in vitro* data establishing that PCBs promote dendritic growth via RyR-dependent mechanisms. The proposal that NDL PCBs contribute to the dendrite-promoting activity of A1254 is strengthened by the observation that developmental exposure to PCB-95 in the maternal diet similarly stimulates dendritic growth *in vivo*. Interestingly, over the dose ranges tested in this study, PCB-95, but not A1254, exhibited an inverted dose–response relationship. Although we cannot rule out the possibility that nonlinearity would also be observed with A1254 at doses > 6 mg/kg/day, the different dose–response relationships observed for A1254 vs. PCB-95 may reflect the fact that A1254 is a mixture of DL and NDL PCBs with varying RyR potency (Kostyniak et al. 2005). Alternatively, up-regulation of cytochrome P450 enzymes by DL PCBs in A1254 could result in different toxicokinetics of NDL PCBs in A1254- vs. PCB-95–exposed animals (Gauger et al. 2007; Giera et al. 2011).

The nonlinear dose response of developmental PCB-95 exposure on dendritic growth *in vivo* is recapitulated *in vitro*. The loss of dendrite-promoting activity *in vitro* at PCB-95 concentrations > 2 μM is likely not due to decreased cell viability (Howard et al. 2003). A plausible explanation is that chronic RyR sensitization alters RyR expression and/or activity, in an inverted concentration-related manner, which is perhaps due to local production of reactive oxygen species (ROS) by PCBs (Fonnum et al. 2006). Microsomal RyR complexes possess a small number of highly reactive cysteines that confer tight regulation of Ca^2+^ channel activity in response to changing transmembrane redox potential (Feng et al. 2000; Liu et al. 1994). Thus the sensitizing and desensitizing influence of RyR-active PCBs on Ca^2+^ signaling may be strongly influenced by the level of ROS production (Pessah et al. 2002). A parallel mechanism has been demonstrated in which moderate increases in Ca^2+^ promote dendritic growth, whereas large increases cause dendritic retraction (Lohmann and Wong 2005; Segal et al. 2000). Thus, when increasing the concentration of PCB-95 from 200 nM to 2 μM, intracellular Ca^2+^ concentrations may cross the threshold from concentrations that promote dendritic growth to those that cause dendritic retraction.

A third possibility is suggested by the report that transfection of cortical neurons with constitutively active CaMKIV (Ca^2+^/calmodulin-dependent protein kinase IV) promotes dendritic growth, whereas expression of constitutively active CaMKII (Ca^2+^/calmodulin-dependent protein kinase II) inhibits dendritic growth (Redmond et al. 2002). Perhaps at higher concentrations that do not promote dendritic growth, PCBs are preferentially activating Ca^2+^-dependent signaling molecules that inhibit dendritic growth. Distinguishing between these possibilities is the focus of future studies.

These data linking a direct molecular effect of NDL PCBs (sensitized RyR activity) to disruption of a specific neurodevelopmental event (dendritic arborization) have significant implications for understanding how PCBs interfere with normal neurodevelopment in the human brain. Structural aberrations in the dendritic arbors of central neurons are thought to contribute to clinical manifestations of diverse heritable and environmentally induced neurodevelopmental disorders in humans (Bourgeron 2009; Fukuda et al. 2005; Garey 2010; Penzes et al. 2011; Svitkina et al. 2010). Consistent with this, developmental A1254 exposure altered dendritic arborization in weanling rats coincident with performance deficits in the Morris swim task (Yang et al. 2009) and exposure to PCB-95 on gestation days 10–16 altered behavior in adult rats (Schantz et al. 1997). NDL PCB congeners with the highest activity towards RyRs, including PCB-95, collectively represent 40–50% of the total PCBs currently found in environmental and biotic samples, and their net effects are likely to be additive (Pessah et al. 2006). However, even low levels of PCB exposure might adversely influence neuronal connectivity in the developing brain of genetically susceptible individuals. Mutations in *RYR* (ryanodine receptor) genes have been linked to environmentally triggered disorders in humans including malignant hyperthermia (Pessah et al. 1996), cardiac arrhythmias (Wehrens et al. 2005), and sudden death (Laitinen et al. 2004). Recent studies demonstrate that specific *RYR* mutations confer sex– and gene–dose-dependent susceptibility to pharmacological (halogenated anesthetic) and environmental (heat) stressors that trigger malignant hyperthermia and muscle damage in otherwise asymptomatic individuals (Barrientos et al. 2012; Yuen et al. 2012). Importantly, PCB-95 is significantly more potent and efficacious in disrupting cation regulation of mutant R615C-*RYR1* compared with wild type RyR1 (Ta and Pessah 2007). Considered together, these observations identify PCBs, and in particular NDL PCBs with high RyR activity, as candidate environmental risk factors in neurodevelopmental disorders and provide important new clues about the possible role of RyRs in contributing to heritable and environmentally triggered neurodevelopmental deficits.

## Supplemental Material

(229 KB) PDF
