# Supplementary material for: PCB-95 Promotes Dendritic Growth via Ryanodine Receptor–Dependent Mechanisms
Source: Environ Health Perspect. 2012 Apr 25;120(7):997–1002. doi: 10.1289/ehp.1104832 (PMC3404670; doi:10.1289/ehp.1104832)
Supplement: (229 KB) PDF [file ehp.1104832.s001.pdf]

Supplemental Material

PCB 95 Induces Dendritic Growth via Ryanodine Receptor-Dependent Mechanisms

Gary A. Wayman[1], Dongren Yang[2], Diptiman D. Bose[2], Adam Lesiak[1], Veronica Ledoux[3], Donald Bruun[2], Isaac N. Pessah[2] and Pamela J. Lein[2,3]*

[1]Program in Neuroscience, Department of Veterinary and Comparative Anatomy, Pharmacology and Physiology, Washington State University, Pullman, WA 99164; [2]Department of Molecular Biosciences, University of California, Davis, CA 95616; [3]Center for Research on Occupational and Environmental Toxicology, Oregon Health & Science University, Portland, OR 97239

***Correspondence should be addressed to:**

Pamela J. Lein, PhD

UC Davis School of Veterinary Medicine, Department of Molecular Biosciences

One Shields Avenue, 1120 Haring Hall, Davis, CA 95616

Telephone: (530) 752-1970; Email: pjlein@ucdavis.edu

Table of Contents

	Page
Supplemental Methods	3
Materials	3
HEK cell culture and transfection	4
Table S1. Sequences of RyR siRNA used in neuronal cells	3
Supplemental References	4
Figure S1**.** Specificity of RyR siRNAs	5

Supplemental Materials and Methods

Materials

Aroclor 1254 (A1254, lot #124-191; purity >99%), PCB 95 (2,2′,3,5′,6-pentachlorobiphenyl, >99% purity) and PCB 66 (2,3',4,4'-tetrachlorobiphenyl, >99% purity) were purchased from AccuStandard, Inc. (New Haven, CT). FLA365 was a generous gift from Michele Chiesi (CIBA). Construction and characterization of the microtubule-associated protein-2B (MAP2B)-enhanced green fluorescent protein (EGFP) fusion construct subcloned into the pCAGGS expression vector and the pCAG-tomato fluorescent protein (TFP) were previously published (Wayman et al. 2006). Three different sets of siRNA primers with differing GC levels were designed for RyR1 and for RyR2 using the Invitrogen (Carlsbad, CA) stealth RNAi service. Each set (which was a mixture of 2 separate siRNAs) was tested for efficiency and specificity using HEK cells stably transfected with either *Ryr1* or *Ryr2*. Data from these experiments was used to select one set against each RyR isoform to use in experiments in neuronal cell cultures (described in Table S1). The Invitrogen negative control kit were used as controls; sequences of these negative control siRNA are proprietary but are confirmed by Invitrogen to not be homologous to any expressed genes in the vertebrate transcriptome.

Supplemental Material, Table 1. Sequences of RyR siRNA used in neuronal cells

**RyR1-specific siRNA**	**RyR2-specific siRNA**
CGAGGAACUUCUACACGCUGCGAUU<br>and<br>AAUCGCAGCGUGUAGAAGUUCCUCG	CAAGCGCAUCGAGAGGGUCUACUUU<br>and<br>AAAGUAGACCCUCUCGAUGCGCUUG

HEK Cell Culture and Transfection

Human embryonic kidney (HEK 293) cells were maintained in DMEM medium supplemented with 2 mM glutamine, 100 μg/ml streptomycin, 100 U/ml penicillin, 1 mM sodium pyruvate, and 10% FBS. Cells were transfected with full-length cDNA for rabbit *Ryr1* or *Ryr2* cloned into the pCI-neo expression vector (Promega, Madison, WI) using Lipofectamine 2000 (Invitrogen) according to the manufacturer's instructions. Stably transfected cells were obtained by Geneticin sulfate (500 μg/ml) selection for 2 wk. RyR1 and RyR2 expression were confirmed by immunocytochemistry using antibody 34C (University of Iowa Hybridoma Bank, Iowa City, IA) or C3-33 (generous gift of Dr. G. Meissner, University of North Carolina), respectively. Cultures were transfected with siRNA (25 pmol) using Lipofectamine RNAiMax (Invitrogen) per the manufacturer's instructions. RyR expression was quantified by in-cell western 48 h after transfection using anti-RyR1 antibody 34C (1:25) or anti-RyR2 antibody C3-33 (1:1000), and counterstaining with the nucleic acid binding dye DraQ5 (LICOR-Biosciences). Cultures were scanned using the LICOR Odyssey infrared imager. Total RyR signal (800 nm) was normalized to DraQ5 signal (700 nm) within the same well. Three wells were analyzed per treatment; significant differences were identified using one-way ANOVA with *post hoc* Newman-Keuls.

Supplemental References

Wayman GA, Impey S, Marks D, Saneyoshi T, Grant WF, Derkach V, et al. 2006. Activity-dependent dendritic arborization mediated by CaM-kinase I activation and enhanced CREB-dependent transcription of Wnt-2. Neuron 50(6):897-909.

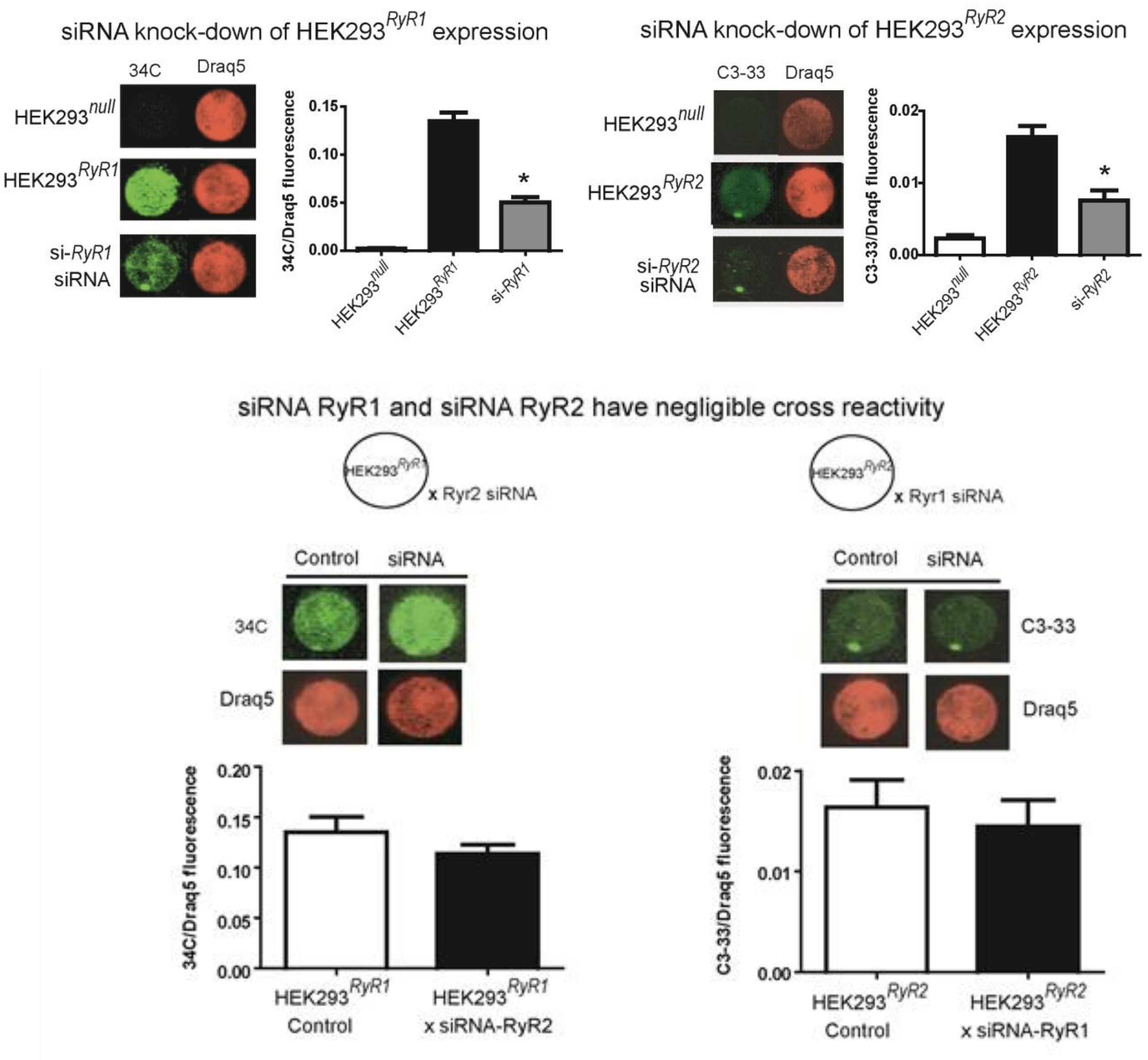

Supplemental Material, Figure 1. Specificity of RyR siRNAs. Wild type HEK293 cell line (HEK293^{null}), which lack detectable RyR expression, were stably transfected with *Ryr1* cDNA (HEK293^{RyR1}, top left) or *Ryr2* cDNA (HEK293^{RyR2}, top right).. RyR expression was quantified via densitometry of in-cell western blots 48 h after transfection with RyR isoform-specific siRNA constructs. Shown are representative images of in-cell western blots using antibodies 34C and C3-33 to probe RyR1 and RyR2, respectively, and Draq5 to stain nuclei; densitometric analyses of RyR1 and RyR2 expression normalized to Draq5 within each well are summarized in the bar graphs. As illustrated in bottom panels, siRNA 2-RyR1 did not knockdown RyR2 expression in HEK293^{RyR2} cells; conversely, siRNA 1-RyR2 did not knockdown RyR1 expression in HEK293^{RyR1} cells. Data expressed as the mean ± SD (n=3 wells).
